# Rethinking expertise in artificial intelligence ethics for global health

**DOI:** 10.1093/inthealth/ihaf114

**Published:** 2025-10-06

**Authors:** Bilal Irfan, Roberto Sirvent

**Affiliations:** Center for Bioethics, Harvard Medical School, Boston, MA 02115, USA; Center for Surgery and Public Health, Brigham & Women’s Hospital, Boston, MA 02120, USA; Department of Neurology, University of Michigan Medical School, Ann Arbor, MI 48109, USA; Department of Epidemiology, University of Michigan School of Public Health, Ann Arbor, MI 48109, USA; Center for Bioethics, Harvard Medical School, Boston, MA 02115, USA; Department of Global Health and Social Medicine, Harvard Medical School, Boston, MA 02115, USA

**Keywords:** algorithmic bias, decolonial ethics, global health, low- and middle-income countries, participatory governance, patient-led oversight

## Abstract

Debates on AI ethics in global health often privilege professionalized authority over those most exposed to harm. We argue for the need to consider redistributing ethical authority to affected communities, particularly in low- and middle-income countries, potentially through participatory councils with decision power over evaluation metrics, equity constraints, and deployment. Centering lived experience can strengthen safety, accountability, and decolonial governance, may aid in addressing algorithmic bias and financial toxicity, and could align with WHO guidance for public-interest AI. We offer potential practical mechanisms to co-lead design, trials, and post-deployment monitoring so AI advances health and rights rather than simply reproducing inequities.

Debates about the ethics of artificial intelligence (AI) usage and in healthcare often begin with questions about stewardship and ascertaining who should be in the room: philosophers, clinicians, economists, data scientists, policymakers and industry leaders. This multidisciplinary roster can be valuable, yet it repeats a familiar pattern in global health by privileging professionalized authority over those who often bear the greatest risks associated with AI use and may have the fewest protections. We argue that ethical authority in AI for health should be recentered around people whose health, safety and survival are most exposed to AI’s harms, especially in low- and middle-income countries (LMICs). Doing so would require more than just a rhetorical regurgitation, but rather a practical strategy to improve safety, accountability and equity in the design, evaluation and governance of AI. It can also meet the WHO’s call for governance arrangements that align AI with public benefit and human rights.^1^

Technical performance or enhancements often cannot be disentangled from the structural power they are rooted in. Digital health agendas frequently run the risk of reproducing colonial logics, concentrating control, capital and data far from the communities whose bodies and behaviors are analyzed.^2^ These dynamics warp problem definitions, shift benefits upstream and externalize risks downstream to patients, workers and marginalized populations. A decolonial approach therefore requires more than ‘inclusion’, it demands a reallocation of epistemic authority to those historically rendered as data sources rather than knowledge producers.

The possibility of inadvertent harm perpetuation from health technologies also provides a basis to consider why affected groups must not be marginal in the decision-making on the implementation of new health tools. For example, a widely used population health algorithm systematically under-rated black American patients’ illness burden because cost was used as a proxy for need, thereby withholding resources from those needing them most.^3^ That is not just some error in implementation, but a failure of problem framing that communities could have flagged had their lived experience guided priority setting and the use of such technologies. Algorithmic bias often emerges across the lifecycle and provides a framework for mitigation that begins with representation, domain knowledge and governance attuned to equity. These analyses can be particularly powerful when they take seriously the perspectives of those on the receiving end of clinical care, health coverage and epidemiological surveillance decisions.^4^

Financial toxicity may also arise when AI tools prompt cascades of confirmatory tests, treatment plans or monitoring that fall outside of insurance plan coverage.^5^ Clinicians and health systems could work to anticipate these gaps, redesign care pathways and press payers for timely coverage alignment so costs are not shifted onto those least able to bear them. The use of AI by insurers and intermediaries for coverage determinations has generated scrutiny and litigation, as automated denials can erode due process and patient trust.^6^ These dynamics support a framework of governance and evaluation co-led by patients and advocates who routinely navigate prior authorization and appeals, because their experience is essential for identifying downstream costs and inequities that administrative and technical perspectives often miss.^7^

Global inequities also shape the landscape of the use of AI in health systems. Many AI efforts remain proof-of-concept in high-income settings, with limited pathways for equitable deployment in LMICs.^8^ Barriers to implementation often include a lack of systematized digital health data infrastructures, shortages in workforce and mismatches between donor priorities and local needs.^8^ In fact, AI can also run the risk of widening inequities when not coupled with investments in local capacity, governance and maintenance.^9^ Placing community health workers, patient advocates, disability organizations, migrant networks and labor organizers at the helm of ethical oversight as more than simply consultees, but rather ones wielding decision-making capability, may be required to address this.^10^

This recentering is also pragmatic for safety and performance. The promise of conversational diagnostic systems and other large multi-modal models (LMMs) is now supported by rapid technical progress, yet even optimistic evaluations often emphasize limits, context-specific sensitivity and the need for real-world trials that measure patient-relevant outcomes and unintended effects. The WHO’s 2025 guidance on LMMs provides a basis to situate that governance must be anticipatory and participatory, with special attention to data provenance, explainability and human control.^1^ If trials and postdeployment monitoring are co-led by people who experience discrimination, precarious work, eviction risk, language barriers or border surveillance, errors that do not usually appear in simulated benchmarks may surface.

The method for centering such expertise is not mysterious. Global health already has playbooks for community governance, including participatory action research that relocates agenda-setting, interpretation and benefit sharing into the community. Applied to AI, this means building standing ethics councils in hospitals and ministries of health where seats are reserved for those most affected by AI-mediated decisions. These councils could control evaluation metrics, define unacceptable risk and choose when to pause or decommission tools. They may also serve to steward data cooperation agreements that negotiate consent, access and value sharing, particularly where cross-border data flows risk a form of data colonialism under the guise of capacity building. All the same, we must cautiously remind that academics too are part of the ruling class (and universities and their form of education are often part of the problem, not the solution), so we must ask what it would like for a group like this to be staffed primarily with people who received their education (and political/social analysis) primarily from outside academia.

Reorienting expertise will also alter what counts as evidence. AI tools may often need to be compared against the health system as it actually functions, not idealized standards.^11^ That is precisely why lived experience matters. Evaluations that ignore these realities could constitute a form of ethical theater where performance metrics obscure distributional consequences. There will be objections. Some will say that technical knowledge must dominate because safety depends on it. The response is to reject a false choice. Technical excellence is necessary, but it is insufficient without moral expertise born from collective struggle against domination in clinics, workplaces and the streets. Others might argue that affected communities can be ‘represented’ by professional advocates. The track record of extractive digital health projects counsels otherwise. When decision power and funding reside with institutions whose incentives are misaligned, often community input is absorbed, translated and neutralized.^2^ Governance that transfers authority can help redirect incentives toward genuine safety and solidarity.

By ‘moral expertise’, we mean practically grounded knowledge of how AI-mediated decisions can translate into burdens, barriers and risks in real lives, expertise that is often acquired through navigating denial of coverage, appeals, debt, disability, language exclusion, border surveillance or occupational hazards. To integrate this expertise, councils could be constituted so that a set number of seats could be reserved for members with lived experience that maps onto the system’s greatest exposure points (e.g. people with disabilities, chronic-care patients subject to prior authorization, migrants and refugees, uninsured or underinsured patients, community health workers and frontline staff). Members could be nominated by community organizations and selected by a transparent lottery to avoid gatekeeping; terms could be staggered; stipends, childcare, language access and accessibility needs may need to be guaranteed; and strict conflict-of-interest rules apply. Technical and policy experts could also sit as non-voting advisors unless explicitly invited to vote by the community bloc. Councils hold for example in some configuration binding authority to: (i) approve ‘ethics release’ before pilot testing and scale-up operating; (ii) set minimum performance and equity constraints (e.g. subgroup calibration, error-rate parity targets, appeal-pathway friction caps, out-of-pocket cost ceilings); (iii) require contract clauses that prevent risk-shifting to patients (audit rights, independent evaluation, remediation funds); and (iv) suspend or sunset systems when predefined harm thresholds are crossed.

Even non-profit organizations that purport to focus on community engagement or cooperation are often made up of academics or people whose primary experience is in the business or tech world (and who may seek recognition from mainstream media about being named the ‘most influential activists’, for example). One possible step an academic working on AI could take is, when feasible and appropriate to the setting, is that whenever they are asked to speak at a panel, to request the host to invite and compensate a member of the community to be a co-speaker. This might encourage, for example, academics working in AI ethics, to be more attuned to mobilizations in their community against data centers being built in sacrifice zones.

Furthermore, it is worth interrogating what is in AI ethics curriculums. Does it include readings, resource guides, analysis and podcasts created by people impacted by AI? Or are all the readings written by academics? We also raise a challenge to scholarly gatekeeping where a peer review article is often seen to intrinsically have more intellectual weight or rigor than a resource guide made by a disability justice organization. There must also be a focus on expanding where the conversations are happening. If they are exclusively nested in university spaces, it can at times be hard for academics to learn about the struggles of members of the community if those places are not accessible to them. It is often important for spaces discussing these issues to build the network necessary to easily invite community members, labor organizers and environmental activists to participate (even to the point that they could make up more than a majority of the contributors).

When it comes to building partnerships, it must be acknowledged that at times community organizations may not want or seek an official partnership with, for example, an elite university, tech company or a bank, because some may feel like their work could be compromised by getting entangled with institutions who too often are the enemy of radical change. There is also a risk that people within movements are bought off, pacified or incorporated into these institutions, which can sometimes serve as a counterinsurgent effect.

Ultimately, AI is a set of tools embedded in institutions shaped by history, capital and power. Ethics can live or die in those institutions. If global health continues to staff AI ethics with the already powerful, we should expect familiar results: gains for some, new burdens for many and widened gaps between them. If, instead, we build ethics infrastructures where those most exposed to harm lead, we stand a chance of realizing the WHO vision of AI that advances public benefit, protects rights and improves health for all.

## Data Availability

No new data were generated or analyzed in support of this research.
